# Supplementary material for: A bifunctional snoRNA with separable activities in guiding rRNA 2’-O-methylation and scaffolding gametogenesis effectors
Source: Nat Commun. 2025 Apr 5;16:3250. doi: 10.1038/s41467-025-58664-y (PMC11971394; doi:10.1038/s41467-025-58664-y)
Supplement: Supplementary file 1 — Supplementary Information [file 41467_2025_58664_MOESM1_ESM.pdf]

Supplementary Information

A bifunctional snoRNA with separable activities in guiding rRNA 2'-O-methylation and scaffolding gametogenesis effectors

Estelle Leroy[1,5], Drice Challal[1,4,5], Stéphane Pelletier[1], Coralie Goncalves[2], Alexandra Menant[1], Virginie Marchand[3], Yan Jaszczyszyn[1], Erwin van Dijk[1], Delphine Naquin[1], Jessica Andreani[1], Yuri Motorin[3], Benoit Palancade[2], Mathieu Rougemaille[1,*]

This PDF file includes:

Supplementary Figures 1 to 6

Supplementary Tables 1 to 4

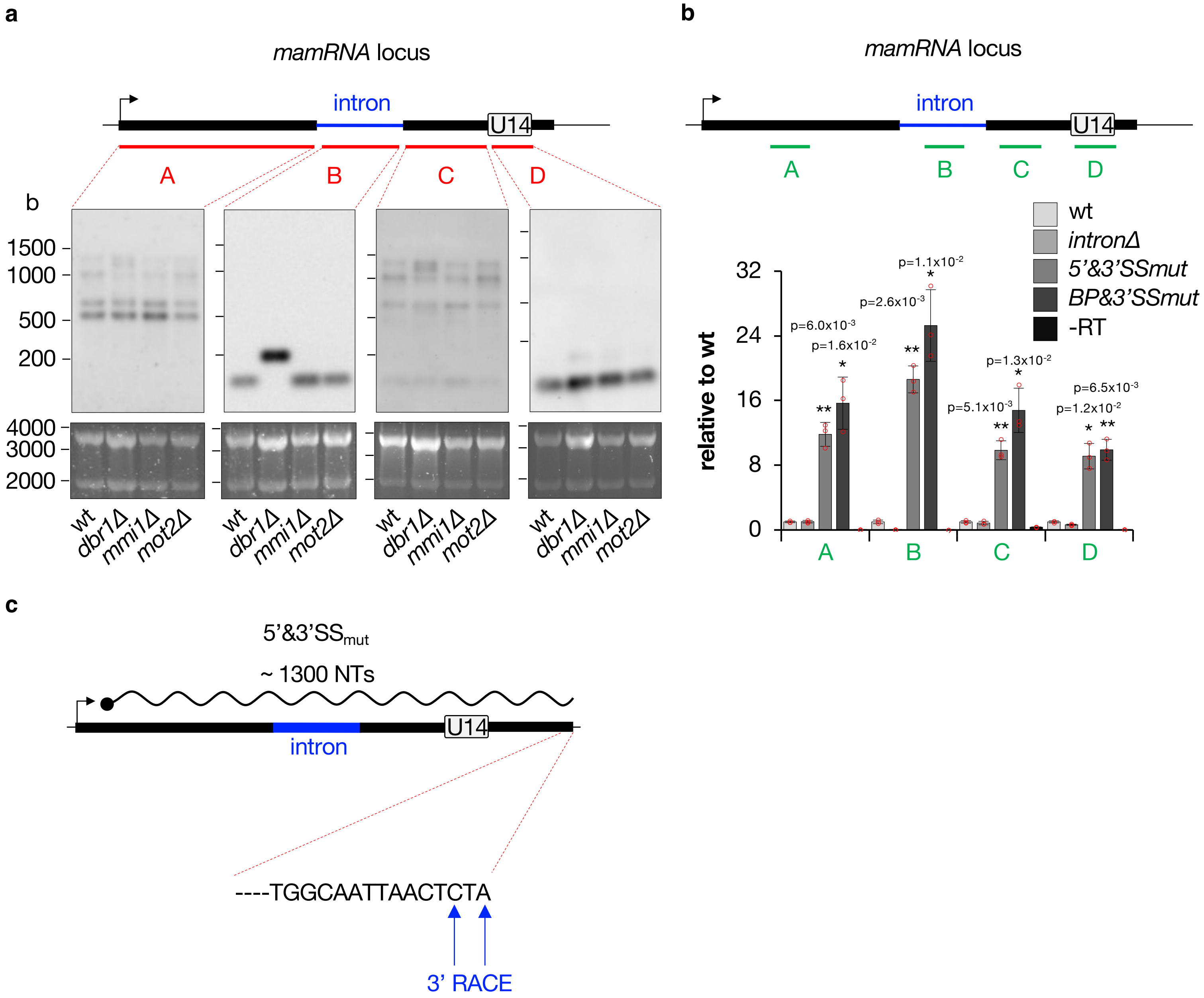

Supplementary Fig. 1 (related to Fig. 1): *mamRNA* is produced from a *U14*-containing precursor and undergoes splicing. a, Northern blots showing *mamRNA* and *U14* levels from total RNA samples, in the indicated genetic backgrounds. The position of the different probes used (A, B, C and D) are depicted on top. Ribosomal RNAs served as loading control (lower panels). b = bases. **b**, RT-qPCR analyses of *mamRNA* and *U14* RNA levels in cells of the indicated genetic backgrounds (mean±SD; n=3 biological replicates; normalized to *act1+* and relative to wt). The position of the different amplicons analyzed (A, B, C, D) is indicated on top. Student's t-test (two-tailed) was used to calculate p-values (relative to wt). Individual data points are represented by red circles. **c**, 3' RACE analysis of the unspliced *mamRNA-U14* transcript in 5'&3'SS_{mut} cells. The sequences of the 3' ends obtained from 2 independent clones are indicated by blue arrows.

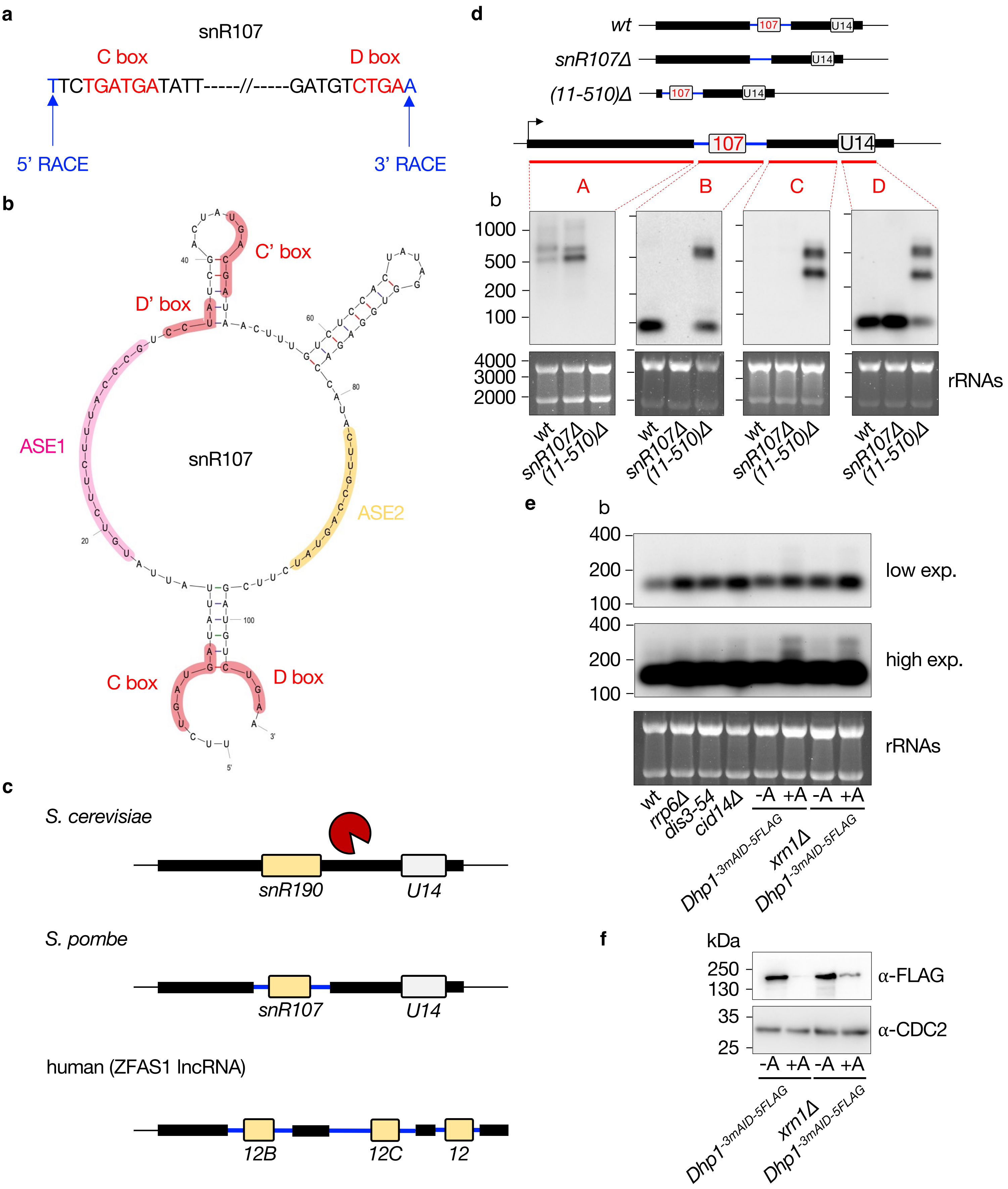

Supplementary Fig. 2 (related to Fig. 2): *mamRNA* intron encodes a C/D-box snoRNA. a, 5' and 3' RACE analyses of *snR107* in wild type cells. The sequences of the 5' and 3' ends were obtained from 1 and 2 independent clones respectively and are indicated by blue arrows. C and D boxes are labeled in red. **b**, Mfold prediction of *snR107* secondary structure. The terminal C and D as well as internal C' and D' boxes are highlighted in red. The ASE1 and ASE2, highlighted in magenta and orange respectively, were maintained single stranded. Note that Mfold does not allow formation of non-canonical A-G base pairs that are commonly found in C/D-box snoRNAs, between the C and D boxes and between the C' and D' boxes, respectively. (continued).

Supplementary Fig. 2 (continued): c, Scheme depicting the genomic organization of *S. cerevisiae snR190*, *S. pombe snR107* and human *SNORD12/B/C*. Blue lines denote intronic sequences. **d**, Northern blots showing *mamRNA* and *snoU14* levels from total RNA samples, in the indicated genetic backgrounds represented on top. The position of the different probes used (A, B, C and D) are depicted. Ribosomal RNAs served as loading control. b = bases. **e**, Northern blots showing *snR107* levels from total RNA samples, in the indicated genetic backgrounds. Ribosomal RNAs served as loading control. An auxin-inducible degron tagged version of Dhp1 was used for protein depletion. -A = no auxin; +A = auxin; b = bases. **f**, Western blots showing the levels of an auxin-inducible degron tagged version of Dhp1 in the indicated genetic backgrounds in the absence (-A) or presence (+A) of auxin. An anti-CDC2 antibody was used as loading control. kDa = kilodaltons.

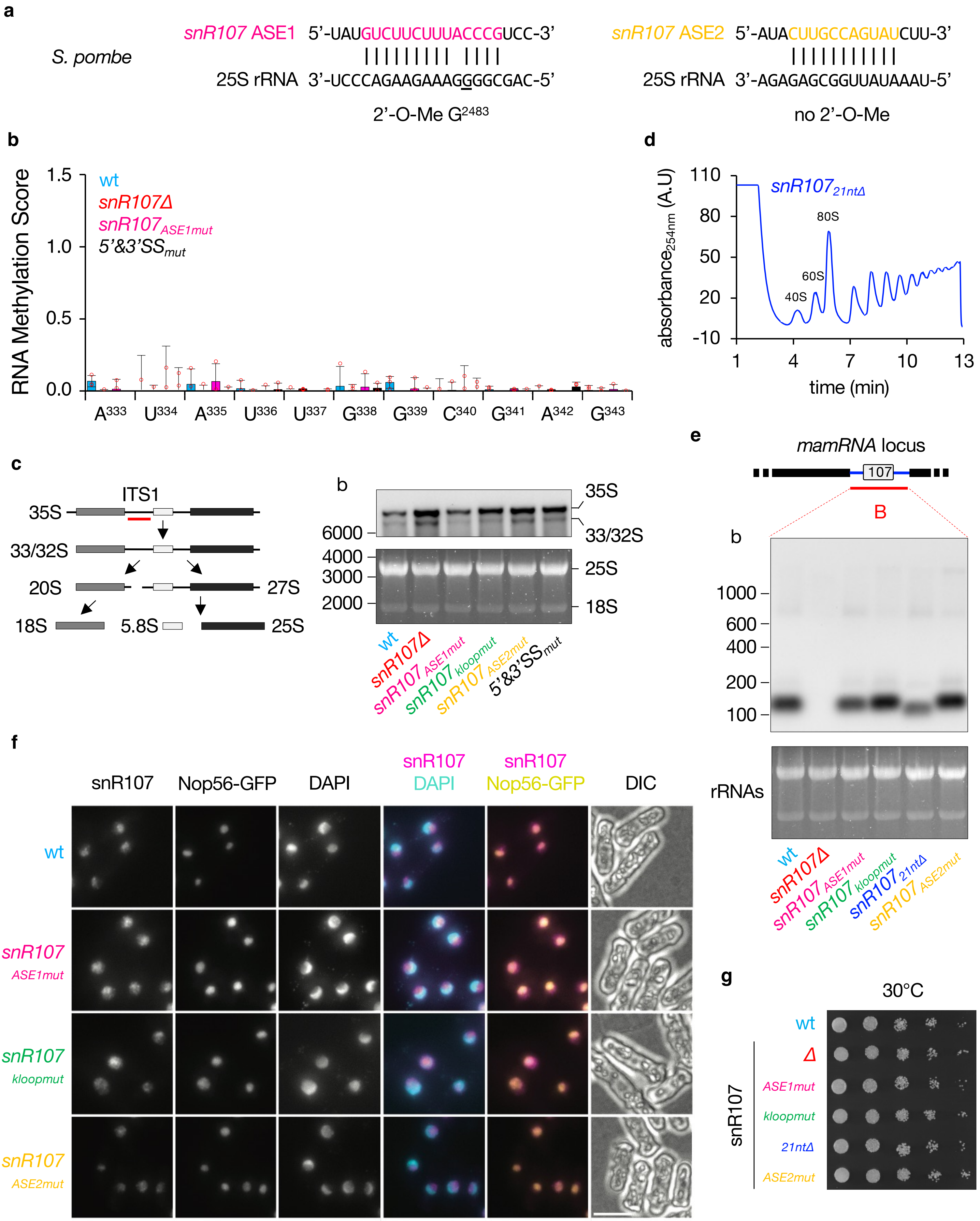

Supplementary Fig. 3 (related to Fig. 3): *snR107* guides 25S rRNA 2'-O-methylation and is required for ribosome biogenesis. a, Scheme showing the sequence complementarities between *snR107* ASE1 (magenta) or ASE2 (orange) and 25S rRNA (black). G^{2483} is underlined. (continued)

Supplementary Fig. 3 (continued): b, RNA methylation scores (RMS) of the 25S rRNA residues 333 to 343 (complementary to *snR107* ASE2) as determined by RiboMethSeq in strains of the indicated genotypes (mean$_{\text{(RMS-median all RMS)}}$±SD; n=3 biological replicates). Individual data points are represented by red circles. **c**, Northern blot showing 35S and 33/32S pre-rRNA levels from total RNA samples, in the indicated genetic backgrounds. The position of the probe is indicated on the left as a red line. Ribosomal RNAs served as loading control. b = bases. **d**, Polysome profiles from total cellular extracts obtained on sucrose gradients in the *snR107*$_{21nt\Delta}$ strain. Absorbance at 254 nm was measured for up to 13 min of collection time and is expressed as arbitrary units (A.U). **e**, Northern blot showing *snR107* levels from total RNA samples, in the indicated genetic backgrounds. Ribosomal RNAs served as loading control. b = bases. **f**, Representative images of *snR107* detected by smFISH in cells expressing GFP-tagged Nop56 in the indicated genetic backgrounds. DNA was stained with DAPI. Images are shown as Z-projections. Scale bar, 5 µm. **g**, 5-fold serial dilution growth assay. Cells of the indicated genotypes were plated on minimal medium (EMM) and grown for 3 days at 30°C.

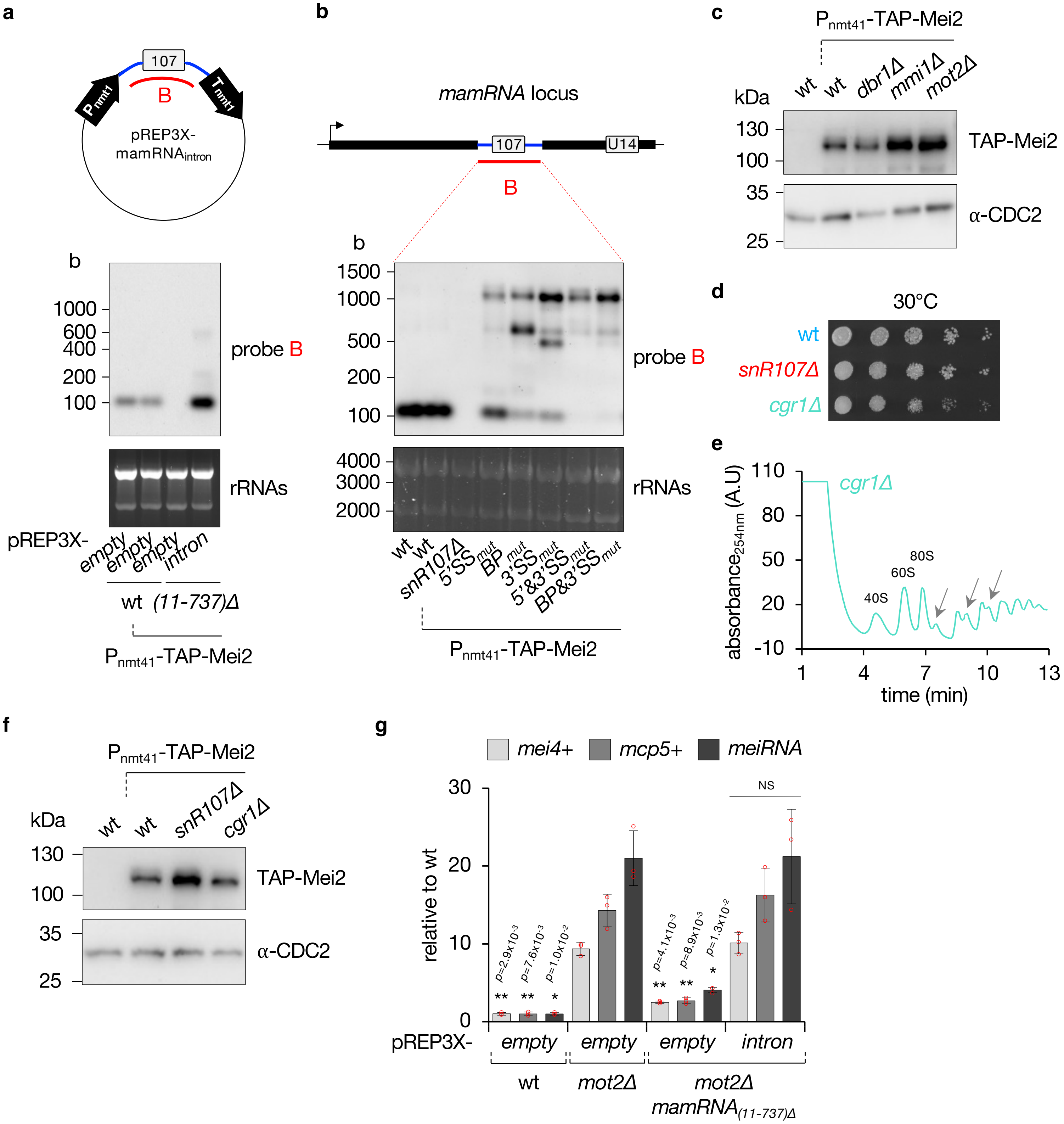

Supplementary Fig. 4 (related to Fig. 4): *snR107* mediates the Mmi1-Mei2 mutual control. a and **b**, Northern blots showing *snR107* levels (probe B) from total RNA samples, in the indicated genetic backgrounds. Ribosomal RNAs served as loading control. b = bases. **c** and **f**, Western blots showing the levels of TAP-tagged Mei2 in cells of the indicated genetic backgrounds. An anti-CDC2 antibody was used as loading control. kDa = kilodaltons. **d**, 5-fold serial dilution growth assay. Cells of the indicated genotypes were plated on minimal medium (EMM) and grown for 3 days at 30°C. **e**, Polysome profiles from total cellular extracts obtained on sucrose gradients in the *cgr1Δ* strain. Absorbance at 254 nm was measured for up to 13 min of collection time and is expressed as arbitrary units (A.U). The grey arrows point to half-mers. **g**, RT-qPCR analyses of *mei4+, mcp5+* meiotic mRNA and *meiRNA* levels in cells of the indicated genetic backgrounds (mean±SD; n=3 biological replicates; normalized to *act1+* and relative to wt). Student's t-test (two-tailed) was used to calculate p-values (relative to *mot2Δ*). Individual data points are represented by red circles.

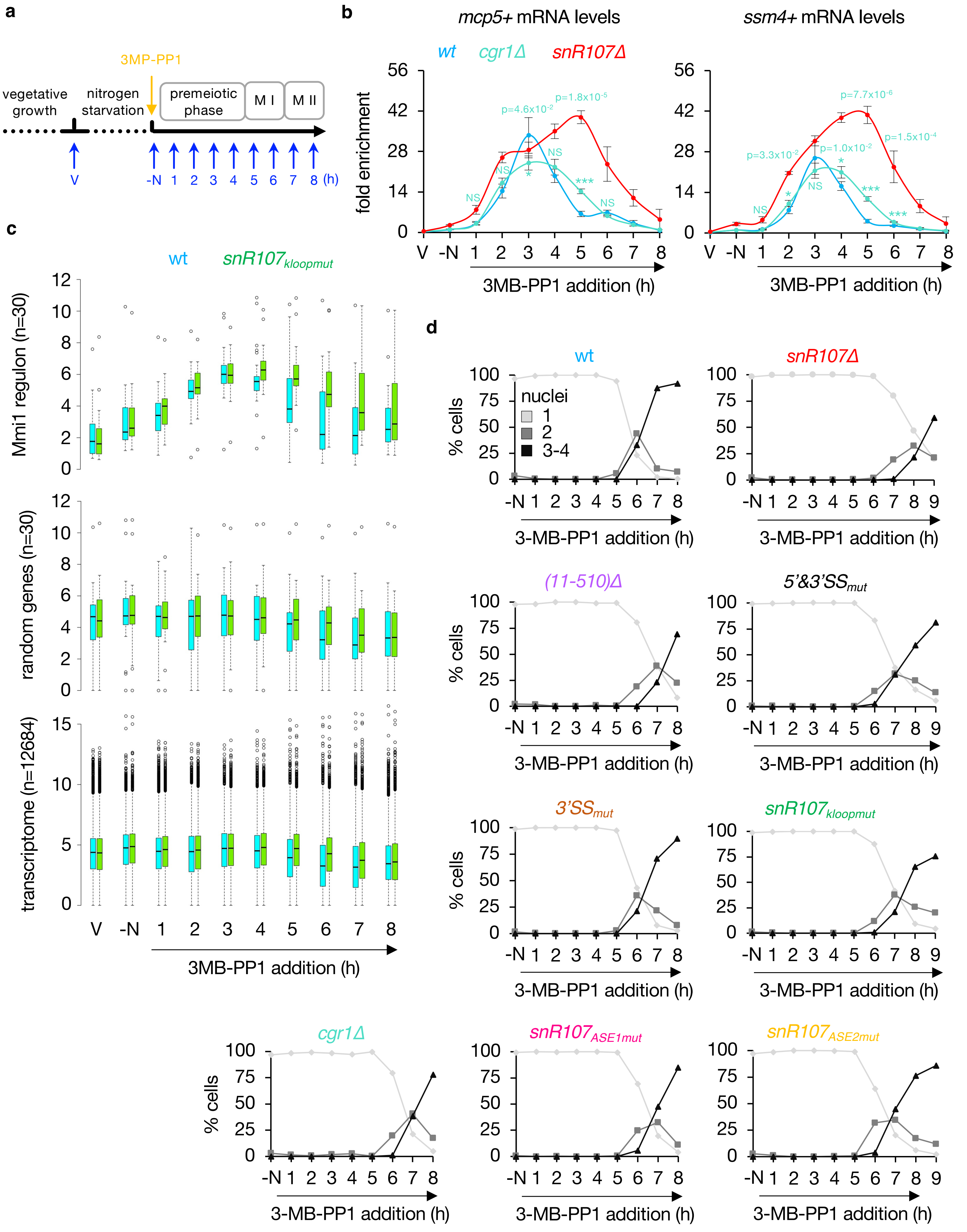

Supplementary Fig. 5 (related to Fig. 5): *snR107* restricts the amplitude and timing of meiotic gene expression.

e

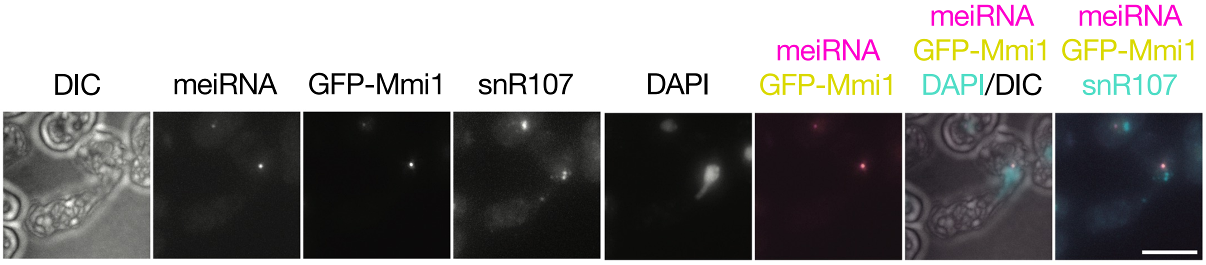

f

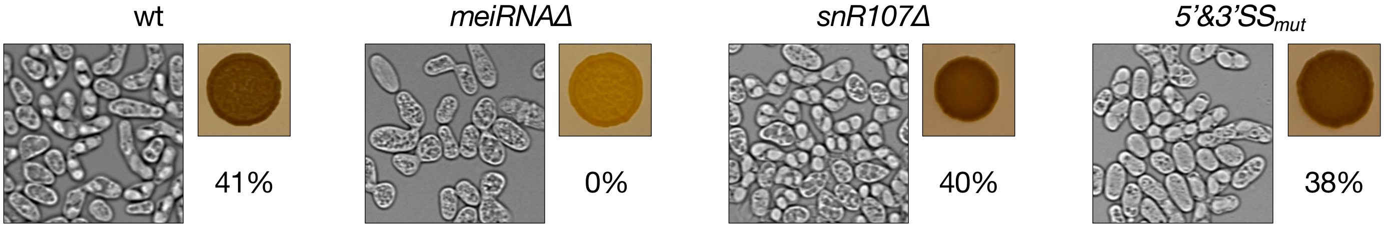

Supplementary Fig. 5 (related to Fig. 5): *snR107* restricts the amplitude and timing of meiotic gene expression. **a**, Scheme illustrating the experimental strategy for the induction of meiosis. Following synchronization in G1 by nitrogen starvation (-N), meiosis was induced by adding 3-MB-PP1 to the culture medium and cells were harvested every hour as indicated. V = vegetative cells; -N = nitrogen-starved cells. **b**, RT-qPCR analyses of *mcp5*+ and *ssm4*+ meiotic mRNA levels upon induction of meiosis in *cgr1Δ* cells (mean±SD; n=4 biological replicates; normalized to total RNA concentration and relative to wt -N). Note that data for the wild type and *snR107Δ* strains in **Fig. 5c** were replotted here to ease comparison. Student's t-test (two-tailed) was used to calculate p-values (relative to wt). NS = not significant. **c**, Comparison of wild type and $snR107_{kloopmut}$ poly(A)+ transcriptomes by RNA-seq following induction of meiosis (n=2). Box plots show the relative expression (log2) of 30 Mmi1 targets[10] (upper panel), 30 random transcripts (middle panel) and the entire transcriptome (n=12684 RNAs). Box center lines represent the median, box limits represent the upper and lower quartiles, whiskers define the 1.5x interquartile range and individual points correspond to outliers. **d**, Meiotic time course in cells of the indicated genotypes. Hoechst-stained nuclei were counted at the indicated time points. Values represent the percentages of cells with one nucleus (diamonds), two nuclei (squares), three or four nuclei (triangles) from two independent experiments. **e**, Representative images of *meiRNA* and *snR107* detected by smFISH in wild type meiotic cells (horsetail stage of meiotic prophase I) expressing GFP-tagged Mmi1. DNA was stained with DAPI. Images are shown as Z-projections. Scale bar, 5 µm. **f**, Mating/sporulation efficiencies of the indicated homothallic strains (% tetrads), as determined by iodine staining and live cell imaging.

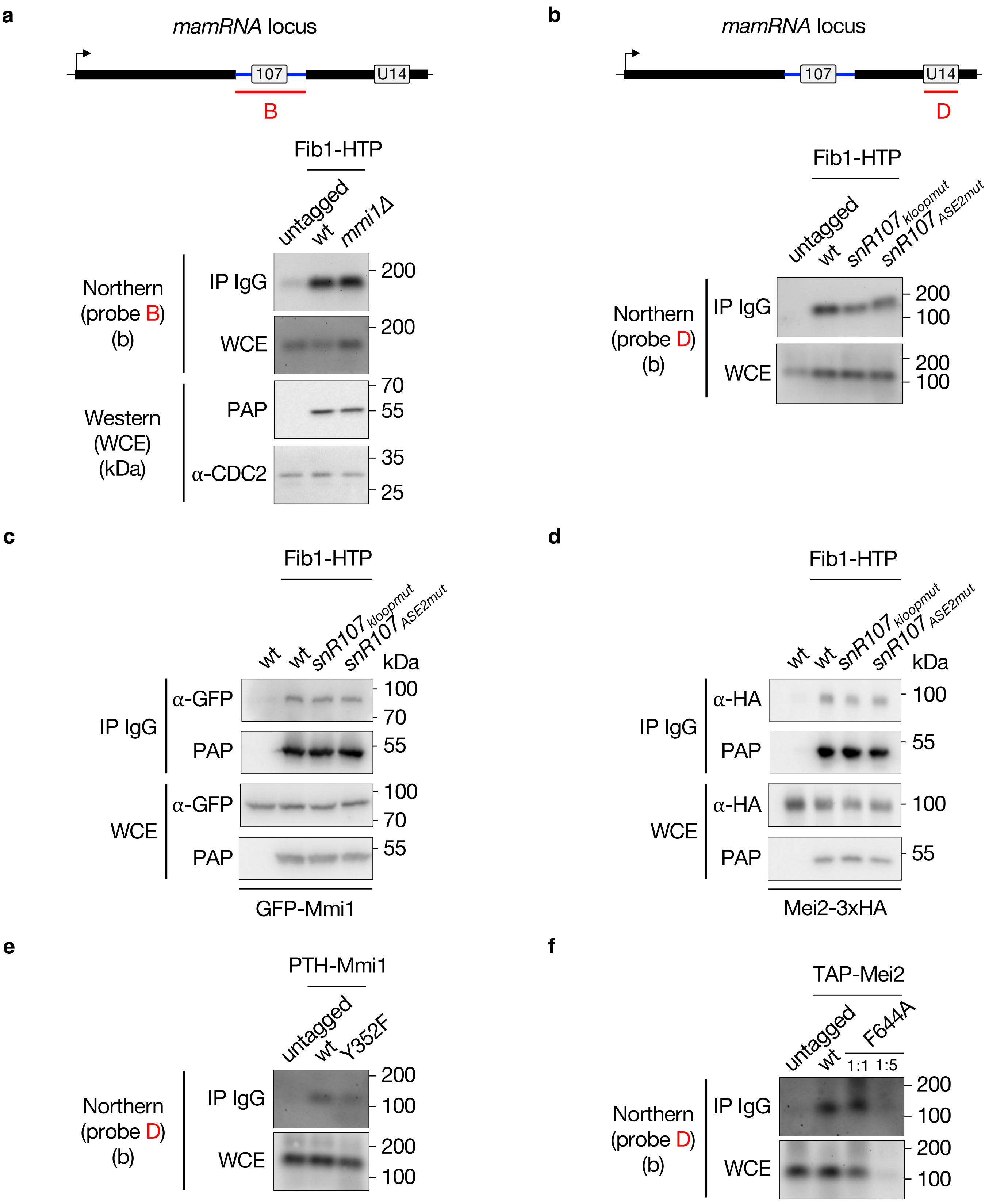

Supplementary Fig. 6 (related to Fig. 6): Mmi1 and Mei2 associate with *snR107* and the 2'-O-methyltransferase Fib1. **a**, **b**, **e** and **f**, Northern blots showing the fractions of *snR107* (probe B) or *U14* (probe D) immunoprecipitated with HTP-tagged Fib1 (**a** and **b**), HTP-tagged Mmi1 (**e**) or TAP-tagged Mei2 (**f**) in cells of the indicated genetic backgrounds. Untagged strains were used as negative controls. IP = Immunoprecipitate; WCE = Whole Cell Extract. (b) = bases. In (**a**), lower panels show Western blotting of total HTP-tagged Fib1 detected with PAP in the corresponding whole cell extracts (WCE). An anti-CDC2 antibody was used as loading control. (kDa) = kilodaltons. **c** and **d**, Western blots showing that GFP-tagged Mmi1 (**b**) and 3xHA-tagged Mei2 (**c**) co-immunoprecipitate with HTP-tagged Fib1 in cells of the indicated genotypes. The Fib1-Mei2 coIP (**c**) was carried out upon meiosis induction, 3 hours post Pat1 inactivation. IP = Immunoprecipitate; WCE = Whole Cell Extract. kDa = kilodaltons.

Supplementary Table 1: *S. pombe* strains used in this study

Strain	Genotype	Source
PR040	h90, *ura4-DS/E, ade6-M210, leu1-32, mat3M::ura4+*	lab stock
PR162	h90, *ura4-D18, ade6-M210, leu1-32, mat3M(EcoRV)::ade6+*	lab stock
PR202	mat3M*::*gfp+*::nat*^{R}*MX*, *leu1-32, ura4-DS/E,* cid14*::hph*^{R}*MX*	This study
PR207	mat3M*::*gfp+*::nat*^{R}*MX*, *leu1-32, ura4-DS/E, his2+/his2-?,* dis3-54	This study
PR486	PR040, rrp6*::hph*^{R}*MX*	Simonetti et al., 2017
PR525	PR162, mot2*::nat*^{R}*MX*	Andric et al., 2021
PR617	PR40, meiRNA*::hph*^{R}*MX*	Andric et al., 2021
PR675	PR40, *kan*^{R}*MX::*P_{nmt41}-TAP-Mei2	Simonetti et al., 2017
PR720	PR40, mot2*::nat*^{R}*MX*, *kan*^{R}*MX::*P_{nmt41}-TAP-Mei2	Simonetti et al., 2017
PR726	PR40, mei4*::nat*^{R}*MX*, mmi1*::hph*^{R}*MX*, *kan*^{R}*MX::*P_{nmt41}-TAP-Mei2	Simonetti et al., 2017
PR1034	PR40, mei2*::hph*^{R}*MX*, pREP41*::LEU2*	Andric et al., 2021
PR1243	PR40, pREP3X*::LEU2*	This study
PR1244	PR40, *kan*^{R}*MX::*P_{nmt41}-TAP-Mei2, pREP3X*::LEU2*	This study
PR1407	PR40, mei2*::hph*^{R}*MX*, pREP41-TAP-Mei2*::LEU2*	Andric et al., 2021
PR1408	PR40, mei2*::hph*^{R}*MX*, pREP41-TAP-Mei2^{F644A}*::LEU2*	Andric et al., 2021
PR1513	M(h-), *leu1-32*, pat1^{L95G}*::kan*^{R}	J. Ayté
PR1609	PR1513, Mei2-HTP*::nat*^{R}*MX*	This study
PR1849	PR1513, mot2*::nat*^{R}*MX*	This study
PR1889	PR1513, mei4*::nat*^{R}*MX*, mmi1*::hygTK*	This study
PR1954	PR1513, mamRNA-intronΔ	This study
PR1956	PR1513, mamRNA$_{5'_splice_site_mutated}$ (5'SSmut)	This study
PR1957	PR1513, mamRNA$_{3'_splice_site_mutated}$ (3'SSmut)	This study
PR1958	PR1513, mamRNA$_{5'_and_3'_splice_sites_mutated}$ (5'&3'SSmut)	This study
PR1968	PR1513, mamRNA$_{(11\text{-}510)\Delta}$	This study
PR1975	PR40, mamRNA$_{intron\Delta}$, *kan*^{R}*MX::*P_{nmt41}-TAP-Mei2	This study
PR1977	PR40, mamRNA$_{5'_splice_site_mutated}$ (5'SSmut), *kan*^{R}*MX::*P_{nmt41}-TAP-Mei2	This study
PR1978	PR40, mamRNA$_{3'_splice_site_mutated}$ (3'SSmut), *kan*^{R}*MX::*P_{nmt41}-TAP-Mei2	This study
PR1979	PR40, mamRNA$_{5'_and_3'_splice_sites_mutated}$ (5'&3'SSmut), *kan*^{R}*MX::*P_{nmt41}-TAP-Mei2	This study
PR1980	PR40, mamRNA$_{(11\text{-}510)\Delta}$, *kan*^{R}*MX::*P_{nmt41}-TAP-Mei2	This study

PR1984	PR40, mamRNA$_{(11\text{-}737)\Delta}$, *kan*^{R}*MX::*P$_{nmt41}$-TAP-Mei2	This study
PR1992	PR40, mamRNA$_{branchpoint_mutated}$ (BPmut), *kan*^{R}*MX::*P$_{nmt41}$-TAP-Mei2	This study
PR2001	PR40, mamRNA$_{5'_and_3'_splice_sites_mutated}$ (5'&3'SSmut)	This study
PR2004	PR1513, P$_{mmi1}$-PTH-Mmi1	This study
PR2012	PR40, dbr1*::hygTK*, *kan*^{R}*MX::*P$_{nmt41}$-TAP-Mei2	This study
PR2013	PR1513, dbr1*::hygTK*	This study
PR2032	PR1513, Fib1-HTP*::nat*^{R}*MX*	This study
PR2033	PR1513, Nop56-HTP*::nat*^{R}*MX*	This study
PR2034	PR1513, Nop58-HTP*::nat*^{R}*MX*	This study
PR2059	PR1513, Fib1-HTP*::nat*^{R}*MX*, Mei2-3xHA*::hph*^{R}*MX*	This study
PR2066	PR1513, mamRNA$_{branchpoint_mutated}$ (BPmut)	This study
PR2067	PR1513, mamRNA$_{branchpoint+3'_splice_site_mutated}$ (BP+3'SSmut)	This study
PR2071	PR1513, Mei2-3xHA*::hph*^{R}*MX*	This study
PR2079	PR40, snR107$_{ASE1mut}$, *kan*^{R}*MX::*P$_{nmt41}$-TAP-Mei2	This study
PR2083	PR40, snR107Δ, *kan*^{R}*MX::*P$_{nmt41}$-TAP-Mei2	This study
PR2097	PR40, snR107$_{21nt\Delta}$, *kan*^{R}*MX::*P$_{nmt41}$-TAP-Mei2	This study
PR2099	PR40, snR107$_{ASE2mut}$, *kan*^{R}*MX::*P$_{nmt41}$-TAP-Mei2	This study
PR2101	PR1513, snR107Δ	This study
PR2102	PR40, P$_{mmi1}$-GFP-Mmi1	This study
PR2106	PR40, snR107$_{kloopmut}$, *kan*^{R}*MX::*P$_{nmt41}$-TAP-Mei2	This study
PR2111	PR40, mamRNA$_{branchpoint+3'_splice_site_mutated}$ (BP+3'SSmut), *kan*^{R}*MX::*P$_{nmt41}$-TAP-Mei2	This study
PR2116	M(h-), *ura4-D18*, ade6::ade6+-P$_{adh15}$-skp1-AtTIR1-2NLS P$_{adh15}$-skp1-OsTIR1, Dhp1-3xmAID-5xFLAG*::kan*^{R}*MX*	YGRC (FY39932)
PR2117	PR1513, snR107$_{kloopmut}$	This study
PR2120	PR1513, snR107$_{ASE1mut}$	This study
PR2121	PR1513, snR107$_{ASE2mut}$	This study
PR2125	PR1513, Snu13-HTP*::nat*^{R}*MX*	This study
PR2129	PR1513, snR107$_{kloopmut}$, Mei2-HTP*::nat*^{R}*MX*	This study
PR2136	PR1513, snR107$_{kloopmut}$, Fib1-HTP*::nat*^{R}*MX*	This study
PR2137	PR1513, snR107$_{ASE2mut}$, Mei2-HTP*::nat*^{R}*MX*	This study
PR2141	PR162, mot2*::nat*^{R}*MX*, snR107Δ	This study
PR2142	PR162, snR107Δ	This study
PR2143	PR162, mot2*::nat*^{R}*MX*, snR107$_{kloopmut}$	This study
PR2144	PR162, mot2*::nat*^{R}*MX*, mamRNA$_{5'_and_3'_splice_sites_mutated}$ (5'&3'SSmut)	This study
PR2145	PR162, mot2*::nat*^{R}*MX*, snR107$_{ASE1mut}$	This study
PR2146	PR162, mot2*::nat*^{R}*MX*, snR107$_{ASE2mut}$	This study
PR2148	M(h-), *ura4-D18*, ade6::ade6+-P$_{adh15}$-skp1-AtTIR1-2NLS P$_{adh15}$-skp1-OsTIR1, Dhp1-3xmAID-5xFLAG*::kan*^{R}*MX*, xrn1*::nat*^{R}*MX*	This study

PR2155	PR1513, snR107$_{kloopmut}$, P$_{mmi1}$-PTH-Mmi1	This study
PR2156	PR1513, snR107$_{ASE2mut}$, P$_{mmi1}$-PTH-Mmi1	This study
PR2158	PR1513, snR107$_{ASE2mut}$, Fib1-HTP*::nat^{R}MX*	This study
PR2159	PR1513, snR107$_{kloopmut}$, Nop58-HTP*::nat^{R}MX*	This study
PR2160	PR1513, snR107$_{ASE2mut}$, Nop58-HTP*::nat^{R}MX*	This study
PR2161	PR162, Nop56-GFP*::kan^{R}MX*	This study
PR2166	PR1513, snR107$_{kloopmut}$, Fib1-HTP*::nat^{R}MX*, Mei2-3xHA*::hph^{R}MX*	This study
PR2167	PR1513, snR107$_{ASE2mut}$, Fib1-HTP*::nat^{R}MX*, Mei2-3xHA*::hph^{R}MX*	This study
PR2175	PR40, mamRNA$_{(11\text{-}510)\Delta}$, P$_{mmi1}$-GFP-Mmi1	This study
PR2176	PR40, mamRNA$_{intron\Delta}$, P$_{mmi1}$-GFP-Mmi1	This study
PR2177	PR40, snR107Δ, P$_{mmi1}$-GFP-Mmi1	This study
PR2178	PR40, mamRNA$_{5'_and_3'_splice_sites_mutated}$ (5'&3'SSmut), P$_{mmi1}$-GFP-Mmi1	This study
PR2181	PR162, snR107$_{ASE2mut}$, Nop56-GFP*::kan^{R}MX*	This study
PR2188	PR40, snR107Δ	This study
PR2190	PR162, snR107$_{kloopmut}$, Nop56-GFP*::kan^{R}MX*	This study
PR2192	PR40, Fib1-HTP*::nat^{R}MX*, P$_{mmi1}$-GFP-Mmi1	This study
PR2193	PR40, snR107$_{kloopmut}$, Fib1-HTP*::nat^{R}MX*, P$_{mmi1}$-GFP-Mmi1	This study
PR2194	PR40, snR107$_{ASE2mut}$, Fib1-HTP*::nat^{R}MX*, P$_{mmi1}$-GFP-Mmi1	This study
PR2196	PR162, mamRNA$_{5'_and_3'_splice_sites_mutated}$, Nop56-GFP*::kan^{R}MX*	This study
PR2205	PR1513, mei4*::nat^{R}MX*, P$_{mmi1}$-PTH-Mmi1^{Y352F}	This study
PR2206	PR162, snR107$_{ASE1mut}$, Nop56-GFP*::kan^{R}MX*	This study
PR2220	PR1513, snR107$_{21nt\Delta}$	This study
PR2221	PR40, Fib1-HTP*::nat^{R}MX*	This study
PR2222	PR40, mei4*::kan^{R}MX*, mmi1*::hygTK*, Fib1-HTP*::nat^{R}MX*	This study
PR2301	PR1513, cgr1*::hygTK*	This study
PR2302	PR40, cgr1*::hygTK*, *kan^{R}MX::*P$_{nmt41}$-TAP-Mei2	This study
PR2315	PR40, mamRNA$_{(11\text{-}737)\Delta}$, *kan^{R}MX::*P$_{nmt41}$-TAP-Mei2, pREP3X*::LEU2*	This study
PR2316	PR40, mamRNA$_{(11\text{-}737)\Delta}$, *kan^{R}MX::*P$_{nmt41}$-TAP-Mei2, pREP3X-mamRNA$_{intron}$*::LEU2*	This study
PR2317	PR162, mot2*::nat^{R}MX*, mamRNA$_{(11\text{-}737)\Delta}$, pREP3X*::LEU2*	This study
PR2318	PR162, mot2*::nat^{R}MX*, mamRNA$_{(11\text{-}737)\Delta}$, pREP3X-mamRNA$_{intron}$*::LEU2*	This study
PR2319	PR162, pREP3X*::LEU2*	This study
PR2320	PR162, mot2*::nat^{R}MX*, pREP3X*::LEU2*	This study

Supplementary Table 2: Plasmids used in this study

Number	Vector	Purpose	Source
B3874	pFA6a-kanMX6, amp^R, ori	gene deletion	Lab stock
B3877	pFA6a-GFP(S65T)-kan^RMX6, amp^R, ori	C-terminal tagging	Lab stock
BHM1296	pFA6a-nat^RMX6, amp^R, ori	gene deletion	Lab stock
BHM1524	pRS316-3xHA-hph^RMX, amp^R, ori	C-terminal tagging	Lab stock
BMR006	pRS316-hph^RMX-P_{act1}-Ers1(first 500 nucleotides), amp^R, ori, URA3, CEN	gene deletion (hph^RMX cassette)	Lab stock
BMR015	pREP41 (P_{nmt41}), LEU2, amp^R, ori	plasmid-driven expression	Lab stock
BMR021	pBS1539-HTP-nat^RMX, amp^R, ori	C-terminal HTP tagging	Lab stock
BMR106	pREP3X (P_{nmt1}), LEU2, amp^R, ori	plasmid-driven expression	Lab stock
BMR193	pREP41-TAP-Mei2 (P_{nmt41}), LEU2, amp^R, ori	plasmid-driven expression	Lab stock
BMR194	pREP41-TAP-$Mei2^{F644A}$ (P_{nmt41}), LEU2, amp^R, ori	plasmid-driven expression	Lab stock
BMR208	pFA6a-HyTkAX	gene deletion	Addgene, #73898
BMR287	pREP3X-$mamRNA_{intron}$ (P_{nmt1}), LEU2, amp^R, ori	plasmid-driven expression	This study

Supplementary Table 3: Oligonucleotides used in this study

Number	Location	Sequence	Assay
P249	*mei4+* fwd	5'-TGGATCAGATCCGTGGAATC-3'	RT-qPCR
P250	*mei4+* rev	5'-AACGCTCGATTAGAAGGCAT-3'	
P253	*act1+* fwd	5'-AACCCTCAGCTTTGGGTCTT-3'	
P254	*act1+* rev	5'-TTTGCATACGATCGGCAATA-3'	
P325	*ssm4+* fwd	5'-ACACAGTTTACGGGATTCTA-3'	
P326	*ssm4+* rev	5'-GATTGTGATGAAAACTGGGT-3'	
P607	*mcp5+* fwd	5'-AGACGTATTCACCTTACCTC-3'	
P608	*mcp5+* rev	5'-GTTTCCCATCATGACATGTT-3'	
P1638	*meiRNA* fwd	5'-GATGAATAGTAGCTTAGATAGTGATAGT-3'	
P574	*meiRNA* rev	5'-GCTTTCAAGGATAACAATGC-3'	
P1208	*mamRNA 5'* fwd (amplicon A)	5'-TGGGATTAAGTCATCTTGGTG-3'	
P1209	*mamRNA 5'* rev (amplicon A)	5'-GTCAAAAAGATACCACAGCA-3'	
P1392	*U14* fwd (amplicon D)	5'-CAGGTGATGAAATTTCCATTG-3'	
P1284	*U14* rev (amplicon D)	5'-CATCCAAAGGAAGGACTATG-3'	
P1859	*between mamRNA intron and U14* fwd (amplicon C)	5'-ACGTGTATGTTCTGTATGGTG-3'	
P1924	*between mamRNA intron and U14* rev (amplicon C)	5'-CCAAAAAGCAATACAGCACTATATATG-3'	
P2117	*snR107* fwd (amplicon B)	5'-ATGTCTTCTTTACCCGTCCTATC-3'	
P2118	*snR107* rev (amplicon B)	5'-CATCGAAGATACTGGCAAGTATG-3'	
P1313	*mamRNA 5'* fwd	5'-TAGGATGGTTCTCCTTCAGG-3'	RT-PCR
P1284	*U14* rev	5'-CATCCAAAGGAAGGACTATG-3'	
P1517	*mamRNA 5'* fwd (probe A)	5'-TCTGAAGGAGCCACATCAA-3'	Northern (T7 promoter-driven RNA probe)
P2101	*mamRNA 5'* rev (probe A)	5'-TAATACGACTCACTATAGGGCAAACAAGTTTAACAAGGCAC-3'	
P2103	*snR107* fwd (probe B)	5'-GTATGTTCGCCGTATGCTG-3'	
P2104	*snR107* rev (probe B)	5'-TAATACGACTCACTATAGGGGAAGTTAGTACACTGAAAGATTATACAC-3'	
P1859	*between mamRNA intron*	5'-ACGTGTATGTTCTGTATGGTG-3'	

	and U14 fwd (probe C)		
P2211	*between mamRNA intron and U14* rev (probe C)	5’-TAATACGACTCACTATAGGGAACCTTCGCCGAACAATCCA-3’	
P1392	*U14* fwd (probeD)	5’-CAGGTGATGAAATTTCCATTG-3’	
P1449	*U14* rev (probeD)	5’-TAATACGACTCACTATAGGGCGTCAGAAAACACCAGCTGC-3’	
P866	*meiRNA* fwd	5’-CGGAATATGCATGCAAGAT-3’	
P1423	*meiRNA* rev	5’-TAATACGACTCACTATAGGGGTTTCAACAATAGTTCAGGTACAGTCG-3’	
P2355	*25S rRNA 3'* fwd	5’-TGATGAAGTGTCGTCGCAATG-3’	
P2356	*25S rRNA 3'* rev	5’-TAATACGACTCACTATAGGGGATACAGGAGAGTAGGAACAAGTC-3’	
P2362	*ITS1* fwd	5’-CTCTTATCCATTTACCTTTCTGTG-3’	
P2363	*ITS1* rev	5’-TAATACGACTCACTATAGGGAGTAATGATATGCTTGGCATGC-3’	
P2251	*snR107* rev	5'-TACAAGGGATCCCATCGAAGATACTGGCAAGTATG-3'	5' RACE (BamHI site)
P2230	*snR107* fwd	5'-GGACGGGTAAAGAAGACATAGCGATATCATCAGAACAGCTTCG-3'	3' RACE (NdeI site)
P2131	*snR107* fwd #2	5'-ACGTTTCATATGACAACGAAGCTGTTCTGATG-3'	
P2375	*downstream of U14* fwd	5'-GTGTTTGGCTTGTAGTTGTAG-3'	
P2376	*downstream of U14* fwd #2	5'-GATAAAGGTTGATTATCATGTTCTCC-3'	
P2357	*25S rRNA RNA/DNA oligonucleotide* *	5’-mUmUmUCCCCmGmCmUmGmAmUmUmUmUmGmCmC-3’	RNAseH cleavage

* mN: 2’-O-methylated nucleotide

Supplementary Table 4: smFISH probes used in this study

Target RNA	Probe	Sequence
mamRNA 5' (Quasar 670)	#1	5'-GTGGCTCCTTCAGACTCATG-3'
	#2	5'-TGGCGTCCTAGCATATTAAT-3'
	#3	5'-TAAACCTCTTGTCTCCACTA-3'
	#4	5'-GCCTTTCAACATCAGATAAT-3'
	#5	5'-GCCATTTACGATAAGAACCT-3'
	#6	5'-AAGAGCCTGAAGGAGAACCA-3'
	#7	5'-ACACAGATACAGGCGAACGC-3'
	#8	5'-ATCAGACTAGAATCTCTCCA-3'
	#9	5'-GCAACCATACACGACAGACA-3'
	#10	5'-ACCAAGATGACTTAATCCCA-3'
	#11	5'-CCAACACAGTACCAATTACT-3'
	#12	5'-AGGAATATGGTAGGTTTTTC-3'
	#13	5'-AAAGATACCACAGCAAGCGC-3'
	#14	5'-ACTAAGCTTTAACACCCAGT-3'
	#15	5'-AGTATGCGTACGGGATTTAC-3'
	#16	5'-TTCAGTTTGAGTTCAAGGTT-3'
snR107 (Quasar 570)	#1	5'-CATAGTCGATAGGACGGGTA-3'
	#2	5'-TCCACCTATAGTGGAGACAA-3'
	#3	5'-AGACATCGAAGATACTGGCA-3'
	#4	5'-ATAGGACGGGTAAAGAAGAC-3'
	#5	5'-GTGGAGACAAAGTTATCGTC-3'
	#6	5'-GCAAGTATGGTCTCCACCTA-3'
	#7	5'-TCGTCATAGTCGATAGGACG-3'
	#8	5'-TACTGGCAAGTATGGTCTCC-3'
*meiRNA** (Quasar 670)	#1	5’-ATACCCACTAAGTCTGTTTA-3’
	#2	5’-CGGCAGAAGATTGACCAACA-3’
	#3	5’-GCATATTCCGTCTTACAATA-3’
	#4	5’-ACCAACTAAAGCGATCTTGC-3’
	#5	5’-GACCATTTCAAAATGTTGCA-3’
	#6	5’-TACCGAATCCAGCTTTTTGA-3’
	#7	5’-CAGAGCTTAGAAGACAAGGT-3’
	#8	5’-TAACTGGACCCCATCAAGAA-3’
	#9	5’-TAAACCAACTTGGGGGTTGG-3’
	#10	5’-TCTAAGCTACTATTCATCCA-3’
	#11	5’-AGTAGATTCCATCAGTCATA-3’
	#12	5’-TGCAGCCAAAAAGTGTACCA-3’
	#13	5’-CATTGTAAGTGCTTTCAAGG-3’
	#14	5’-TTCAGTCATTCGCAAAGTTT-3’

	#15	5’-AGTCGTTTTATTTCTTTTCT-3’
	#16	5’-GTTTCAACAATAGTTCAGGT-3’
	#17	5’-TCTGTTTCAGGAATACGTTT-3’
	#18	5’-TGTTTCGCATCAAACTTTCA-3’
	#19	5’-GCGTTTAAACAAACTGCGGG-3’
	#20	5’-TGGTTTCAGCACGTTTTCAA-3’
	#21	5’-TTGGTTTGCAGGGTTTAACG-3’
	#22	5’-CTTGCTGTGGTTATTGTTTA-3’

* same probe set as in Andric et al., 2021
