# Supplementary material for: A bifunctional snoRNA with separable activities in guiding rRNA 2’-O-methylation and scaffolding gametogenesis effectors
Source: Nat Commun. 2025 Apr 5;16:3250. doi: 10.1038/s41467-025-58664-y (PMC11971394; doi:10.1038/s41467-025-58664-y)
Supplement: Supplementary file 2 — Description of Additional Supplementary Files [file 41467_2025_58664_MOESM2_ESM.pdf]

Description of Additional Supplementary Files

File name: Supplementary Data 1

Description: rRNA 2'-O-Me residues (related to Fig. 3). Shown are the 18S, 25S and 5.8S 2'-O-methylated residues in the *wt*, *snR107*Δ, *snR107*$_{ASE1mut}$ and *5'&3'SS*$_{mut}$ *S. pombe* strains (RNA Methylation Scores > 0.8 in at least one replicate), as well as their conservation in the budding yeast *Saccharomyces cerevisiae*. G^{2483} is denoted in red.
