# Supplementary material for: A bifunctional snoRNA with separable activities in guiding rRNA 2’-O-methylation and scaffolding gametogenesis effectors
Source: Nat Commun. 2025 Apr 5;16:3250. doi: 10.1038/s41467-025-58664-y (PMC11971394; doi:10.1038/s41467-025-58664-y)
Supplement: Supplementary file 4 — Reporting Summary [file 41467_2025_58664_MOESM4_ESM.pdf]

nature portfolio

Corresponding author(s): Mathieu Rougemaille

Last updated by author(s): Mar 17, 2025

Reporting Summary

Nature Portfolio wishes to improve the reproducibility of the work that we publish. This form provides structure for consistency and transparency in reporting. For further information on Nature Portfolio policies, see our Editorial Policies and the Editorial Policy Checklist.

Statistics

For all statistical analyses, confirm that the following items are present in the figure legend, table legend, main text, or Methods section.

n/a	Confirmed	
☐	☒	The exact sample size (n) for each experimental group/condition, given as a discrete number and unit of measurement
☐	☒	A statement on whether measurements were taken from distinct samples or whether the same sample was measured repeatedly
☐	☒	The statistical test(s) used AND whether they are one- or two-sided<br>*Only common tests should be described solely by name; describe more complex techniques in the Methods section.*
☒	☐	A description of all covariates tested
☒	☐	A description of any assumptions or corrections, such as tests of normality and adjustment for multiple comparisons
☐	☒	A full description of the statistical parameters including central tendency (e.g. means) or other basic estimates (e.g. regression coefficient) AND variation (e.g. standard deviation) or associated estimates of uncertainty (e.g. confidence intervals)
☐	☒	For null hypothesis testing, the test statistic (e.g. F, t, r) with confidence intervals, effect sizes, degrees of freedom and P value noted<br>*Give P values as exact values whenever suitable.*
☒	☐	For Bayesian analysis, information on the choice of priors and Markov chain Monte Carlo settings
☒	☐	For hierarchical and complex designs, identification of the appropriate level for tests and full reporting of outcomes
☒	☐	Estimates of effect sizes (e.g. Cohen's d, Pearson's r), indicating how they were calculated

Our web collection on statistics for biologists contains articles on many of the points above.

Software and code

Policy information about availability of computer code

Data collection	Illumina NextSeq550 and NovaSeq 6000 (Novogene) instruments were used to collect total and polyA+ RNA-seq datasets, respectively.<br>Illumina NextSeq2000 instrument was used to collect RiboMethSeq datasets.<br>PromethION RNA flowcells on a P2 solo instrument were used to collect direct RNA-seq datasets.<br>ChemiDoc MP Imaging System (BIORAD) was used to collect Western blot and Northern blot images.<br>LightCycler LC480 apparatus (Roche) was used to collect qPCR data.<br>Teledyne Isco device equipped with a Tris peristaltic pump and a UA-6 UV/VIS Detector with type 11 optical unit was used to collect polysome profiling data.<br>Motorized Olympus BX63 upright fluorescence microscope equipped with a 60X and 100X oil immersion objective (Olympus), a digital camera C11440 (ORCA-Flash4.0 LT PLUS; Hamamatsu) was used to collect live and fixed cell images.<br>Leica DM6000B microscope equipped with a 100X, NA 1.4 (HCX Plan-Apo) oil immersion objective, a piezo-electric motor (LVDT; Physik Instrument) and a CCD camera (CoolSNAP HQ; Photometrics) was used to collect smFISH images.
Data analysis	bcl2fastq2 v2.18.12 / bcl2fastq v2.20 (demultiplexing), Cutadapt v1.15 / fastp v0.23.1 (trimming), STAR v2.6.1d (alignment), featureCounts from subread v2.0.6 / edgeR (quantification) were used to analyze total and polyA+ RNA-seq datasets.<br>Trimmomatic v0.39 (trimming) and bowtie2 v2.4.4 (alignment) were used for RiboMethSeq analyses.<br>dorado 7.2.13-1 (base-calling) and minimap2 (mapping) were used for direct RNA-seq analyses.<br>Fiji (NIH) and Metamorph (Molecular Devices) were used to analyze live/fixed cell and smFISH images, respectively.

For manuscripts utilizing custom algorithms or software that are central to the research but not yet described in published literature, software must be made available to editors and reviewers. We strongly encourage code deposition in a community repository (e.g. GitHub). See the Nature Portfolio guidelines for submitting code & software for further information.

Data

Policy information about availability of data

All manuscripts must include a data availability statement. This statement should provide the following information, where applicable:

- Accession codes, unique identifiers, or web links for publicly available datasets
- A description of any restrictions on data availability
- For clinical datasets or third party data, please ensure that the statement adheres to our policy

The RNA-seq and dRNA-seq data generated in this study have been deposited in the Gene Expression Omnibus database under accession codes GSE276242 (https://www.ncbi.nlm.nih.gov/geo/query/acc.cgi?acc=GSE276242), GSE276243 (https://www.ncbi.nlm.nih.gov/geo/query/acc.cgi?acc=GSE276243) and GSE276244 (https://www.ncbi.nlm.nih.gov/geo/query/acc.cgi?acc=GSE276244).
The RiboMethSeq data generated in this study have been deposited in the European Nucleotide Archive database under accession code PRJEB79915 (https://www.ebi.ac.uk/ena/browser/view/PRJEB79915).
Source data are provided with this paper.

Research involving human participants, their data, or biological material

Policy information about studies with human participants or human data. See also policy information about sex, gender (identity/presentation), and sexual orientation and race, ethnicity and racism.

Reporting on sex and gender	N/A
Reporting on race, ethnicity, or other socially relevant groupings	N/A
Population characteristics	N/A
Recruitment	N/A
Ethics oversight	N/A

Note that full information on the approval of the study protocol must also be provided in the manuscript.

Field-specific reporting

Please select the one below that is the best fit for your research. If you are not sure, read the appropriate sections before making your selection.

[x] Life sciences [ ] Behavioural & social sciences [ ] Ecological, evolutionary & environmental sciences

For a reference copy of the document with all sections, see nature.com/documents/nr-reporting-summary-flat.pdf

Life sciences study design

All studies must disclose on these points even when the disclosure is negative.

Sample size	No statistical methods were used to estimate sample sizes. n values were chosen in accordance with standard practices and correspond to the number of biological replicates (i.e. independent yeast cultures).
Data exclusions	No data was excluded.
Replication	All experiments were repeated at least two times and different techniques were used to validate findings. Replicates successfully gave similar results.
Randomization	Not relevant. The study focuses on the identification and functional characterization of a lncRNA-encoded snoRNA.
Blinding	Not relevant. The study focuses on the identification and functional characterization of a lncRNA-encoded snoRNA.

Reporting for specific materials, systems and methods

We require information from authors about some types of materials, experimental systems and methods used in many studies. Here, indicate whether each material, system or method listed is relevant to your study. If you are not sure if a list item applies to your research, read the appropriate section before selecting a response.

Materials & experimental systems

n/a	Involved in the study	
☐	☒	Antibodies
☒	☐	Eukaryotic cell lines
☒	☐	Palaeontology and archaeology
☒	☐	Animals and other organisms
☒	☐	Clinical data
☒	☐	Dual use research of concern
☒	☐	Plants

Methods

n/a	Involved in the study	
☒	☐	ChIP-seq
☒	☐	Flow cytometry
☒	☐	MRI-based neuroimaging

Antibodies

Antibodies used

Standard immunoblotting procedures involved peroxydase-conjugated antiperoxydase (Sigma, #P1291, RRID:AB_1079562), anti-CDC2 monoclonal antibody (Abcam, #ab5467, RRID:AB_2074778), anti-FLAG monoclonal antibody (Sigma, #F3165, RRID:AB_259529), anti-GFP monoclonal antibody (Roche, # 11814460001, RRID: AB_390913), anti-HA monoclonal antibody (12CA5) (Sigma, #11583816001, RRID:AB_514505), goat anti-mouse IgG-HRP (Santa Cruz Biotechnology, #sc-2005, RRID:AB_631736).

Validation

All antibodies were previously validated (see manufacturer's websites or references in the Methods section).

Plants

Seed stocks

N/A

Novel plant genotypes

N/A

Authentication

N/A
